# Prognosis and ICU outcome of systemic vasculitis

**DOI:** 10.1186/1471-2253-13-27

**Published:** 2013-10-01

**Authors:** Patrice Befort, Philippe Corne, Thomas Filleron, Boris Jung, Christian Bengler, Olivier Jonquet, Kada Klouche

**Affiliations:** 1 Department of Intensive Care Unit, Gui de Chauliac University Hospital, Montpellier, France; 2 Statistics Claudius Regaud Institute, 20-24 rue du Pont Saint Pierre, Toulouse, France; 3 Department of Anesthesia and Intensive care, Saint Eloi University Hospital, Montpellier, France; 4 Department of Intensive Care, University Hospital, Place Professeur Robert Debré, Nîmes, France; 5 Department of Intensive Care Unit, Lapeyronie University Hospital, 191 Avenue du Doyen G. Giraud, Montpellier 34090, France

**Keywords:** Vasculitis, Outcome, Mortality, Intensive care unit, BVAS

## Abstract

**Background:**

Systemic vasculitis may cause life threatening complications requiring admission to an intensive care unit (ICU). The aim of this study was to evaluate outcomes of systemic vasculitis patients admitted to the ICU and to identify prognosis factors.

**Methods:**

During a ten-year period, records of 31 adult patients with systemic vasculitis admitted to ICUs (median age: 63 y.o, sex ratio M/F: 21/10, SAPS II: 40) were reviewed including clinical and biological parameters, use of mechanical ventilation, catecholamine or/and dialysis support. Mortality was assessed and data were analyzed to identify predictive factors of outcome.

**Results:**

Causes of ICU admissions were active manifestation of vasculitis (n = 19), septic shock (n = 8) and miscellaneous (n = 4). Sixteen patients (52%) died in ICU. By univariate analysis, mortality was associated with higher SOFA (p = 0.006) and SAPS II (p = 0.004) scores. The need for a catecholamine support or/and a renal replacement therapy, and the occurrence of an ARDS significantly worsen the prognosis. By multivariate analysis, only SAPS II (Odd ratio: 1.16, 95% CI [1.01; 1.33]) and BVAS scores (Odd ratio: 1.16, 95% CI = [1.01; 1.34]) were predictive of mortality.

**Conclusion:**

The mortality rate of severe vasculitis requiring an admission to ICU was high. High levels of SAPS II and BVAS scores at admission were predictive of mortality.

## Background

The systemic vasculitides are rare diseases defined by vessel inflammation and classified according to the size of the vessels implicated (Chapel Hill conference) [1,2]. They share clinical features including fever, arthralgia, respiratory distress, renal dysfunction, and neurologic disorders; but their course and prognosis are heterogeneous. Though the use of corticotherapy at 50′s and immunosuppressive therapy at the 70′s significantly improved the outcome with an increase in 5-year survival rate from 20% to 75% [3], they may lead to life-threatening complications requiring admission to Intensive Care Unit (ICU). These severe complications associated with organ failure are mainly related to active diseases or to post-immunosuppressive therapy effects mostly infections [4,5].

Few studies focusing on outcome and prognosis of patients with vasculitides admitted to ICU have been published. Most of the reports included systemic rheumatic diseases like connectivitis, rheumatoïd polyarthritis, granulomatosis, primary and systemic vasculitides [6-13]. High mortality rates (30-50%) were observed in such patients [8,14]. To the best of our knowledge, only *3* studies, the largest one including 38 patients, specifically investigated vasculitides ICU outcome [6,7,13]. They reported a lower in-ICU mortality varying from 11% to 33%. However, a higher mortality rate may occur in case of pulmonary determinations reaching more than 50% [15]. Prognostic systems developed to predict outcomes in critical illness [16,17] were evaluated in such patients as well as the level of vasculitis disease activity. Results showed that severity scores, APACHE II and/or SAPS II, seem to be good predictors of ICU mortality by contrast to the severity of the underlying vasculitis [6,7].

The significant advances, in the past decade, regarding diagnostic strategies and therapeutic options for vasculitis patients led to improvement in overall outcome but also to the use of a more aggressive specific therapy. Thus, ICU-mortality rate and accurate prediction of patients’ outcome upon ICU admission need to be reassessed.

We therefore carried out a ten-year analysis of a cohort of vasculitis patients admitted to ICU in order to investigate the epidemiology, clinical features and outcome of their severe complications, and to identify the predictive factors of mortality.

## Methods

This retrospective study was carried out from 2000 to 2010 at Montpellier and Nîmes University hospitals. All adult patients with systemic vasculitis admitted to four Intensive Care Units (ICUs) (3 medical and 1 medico-surgical ICUs) with a length of stay above 24 hours were included in the study. They were identified from our local informatics’ database. Our local institutional Review Board (Comité de Protection des Personnes Sud Mediterranee IV, Q-2013-09-03) approved the study and waived the need for informed consent.

Vasculitis was classified according to the Chapel Hill conference [2] or to the American College of Rhumatology definitions [18] regarding polyarteritis nodosa, Granulomatosis with polyangiitis (Wegener’s granulomatosis) and Eosinophilic Granulomatosis with polyangiitis (Churg-Strauss). When a patient was hospitalized twice or more, the only first ICU admission was considered. Patients with connective tissue disease (lupus, polyarteritis rheumatoid, others) or primary focal vasculitis (cerebral vasculitis) were excluded. Clinical charts were retrospectively reviewed. Age, sex, and immunosuppressive treatment (above 20 mg daily corticosteroid or/and Cyclophosphamide or Aziathioprine during the 3 months preceding ICU admission) were collected. None of the included patients received biologics like rituximab or infliximab … Upon ICU admission, the reasons for admission were noted and the clinical and biological variables monitored. The severity of the disease was assessed 24 h after admission using the simplified acute physiology score (SAPS) II [16] and the sequential organ failure assessment (SOFA) scores [17] and by the Birmingham Vasculitis Activity Score (BVAS) [19]. BVAS is a clinical index of vasculitis activity based on symptoms and signs in nine separate organ systems (*systemic signs; skin; mucous membranes and eyes; ear; nose and throat; chest; heart and vessels; gastrointestinal tract; kidney; and nervous system*). Presence of a sepsis [20] and source and microorganisms when identified, the occurrence of an acute respiratory distress syndrome (ARDS) [21] or/and a pneumo-renal syndrome [22] were recorded.

During ICU stay, the need for mechanical ventilation, or/and dialysis, or/and vasoactive drugs and their duration use were monitored. ICU length of stay was recorded as well as outcome at ICU discharge. ICU outcome was compared to a control group of matched non-vasculitis ICU patients. The control ICU patients were extracted from our own database and they were frequency matched to cases by age, SAPS II and SOFA scores. One year outcome of ICU survivors was established by phone call or mail interrogating them or their next of kin.

We divided our patients according to ICU mortality and compared survivors and nonsurvivors for all the above variables in order to identify the predictive factors of outcome.

### Statistical analysis

The statistical analyses were performed using STATA 11.0 (StataCorp LP, Texas 77845, USA). We first performed a descriptive analysis by computing the frequencies and the percents for categorial data, median and range for continuous data. To analyze the factors associated with the ICU mortality, univariate and multivariate analyses were performed. The univariate analysis was performed using two-tailed Mann–Whitney test for quantitative variables and Pearson’s Chi-2 test or Fisher’s exact test for qualitative variables when appropriate. Multivariate analysis was performed by logistic regression in order to evaluate the adjusted effects of the different variables. We built the final multivariate model using a backward strategy with all clinical variables measured before or at the admission in the ICU having a p-value less than 10% in univariate analysis. At each step, the variable with the least significant p value was removed until all remaining main effects in the model were significant at p < 0.05. A long-term survival curve was generated using the Kaplan-Meier methodology. A value of p < 0.05 was considered as significant.

## Results

### Population and mortality

Thirty-one patients, ten females and twenty-one males, were included in the study. Patient characteristics are summarized in Table 1. Vasculitis types included Granulomatosis with polyangiitis (n = 15), polyarteritis nodosa (n = 4), IgA vasculitis (n = 4), cryoglobulinemia (n = 3), Giant cell angiitis (n = 2), Eosinophilic Granulomatosis with polyangiitis (n =1), and microscopic polyangiitis (n = 2). Twenty-two patients with a previous established diagnosis of vasculitis received a corticotherapy and 10 of them received an immunosuppressive treatment (Cyclophosphamide or Aziathioprine) in the previous 3 months. Vasculitis was diagnosed during ICU stay in 7 cases (23%): Granulomatosis with polyangiitis n = 5, Eosinophilic Granulomatosis with polyangiitis n = 1 and microscopic polyangiitis n = 1.

**Table 1:** Patients characteristics

**Characteristics**	
**Age,** years	*63 (29–86)*
**Male/female,** n	*21/10*
**Corticotherapy before admission,** n	
Yes	*22*
No	*9*
**Comorbidities**	
Ischemic cardiopathy	*4*
Chronic renal failure	*4*
Chronic respiratory failure	*2*
Non Insulin dependent diabetes	*4*
**Creatininemia,** μmol/l	*232 (32–1067)*
**SAPS II**	*40 (13–109)*
**SOFA**	*5,5 (1–14)*
**BVAS**	*22 (2–52)*
**ICU length of stay,** days	*14 (1–113)*
**In hospital length of stay,** days	*39 (6–288)*
**ICU mortality,** n (%)	*16 (52%)*

Data are presented as median (range), otherwise specified.

*SAPSII* simplified acute physiology score II*, SOFA* sequential organ failure assessment*, BVAS* Birmingham Vasculitis Activity Score*, ICU severity scores were assessed 24 hours after ICU admission.*

Main reasons for ICU admission were active vasculitis (19 patients, 61%) including pneumo-renal syndrome (n = 9), pulmonary haemorrhage (n = 4), cardiogenic shock (n = 2), digestive and renal involvement in Iga vasculitis (n = 2), acute renal failure associated with distal arterial ischemia during polyarteritis nodosa (n = 1), central neurologic involvement in polyarteritis nodosa (n = 1). Eight (26%) patients were admitted for septic shock, and four (13%) for other reasons: subdural hematoma on anticoagulant therapy (n = 1), haemorrhagic shock following hepatic biopsy (n = 1), severe pulmonary embolism (n = 1), and severe post-surgery complication (n = 1). As shown in Table 1, high median SAPS II and SOFA scores underlined the severity of the patient illnesses. During ICU stay, specific vasculitis treatment included corticosteroids in 28 patients, Cyclophosphamide in 12, plasma exchange in 6 and intravenous immunoglobulins in 6 patients. Any patient may received one or more specific treatment. Twenty-five patients (81%) were on mechanical ventilation, 18 (58%) underwent hemodialysis, and 14 (45%) required a catecholamine support. ICU therapeutic management is displayed in Table 2. Nosocomial or secondary infections occurred frequently: 8 ventilator associated pneumonia (Escherichia coli 1, Enterobacter aerogenes 1, Enterobacter cloacae 1, Xanthomonas maltophilia 1, Pseudomonas aeruginosa 1, Staphylococcus aureus 1, Mycobacterium xenopi 1, Streptococcus pneumoniae 1), 5 bacteraemia (Staphylococcus aureus 3, Corynebacterium jekium 1, Anaerobia 1), 5 viral cytomegalovirus infections (pneumonia 2, colitis 1, oesophagitis 1, cutaneous ulceration 1), and 2 mycotic infections (Parapsilosis candidemia 1, Aspergillus fumigatus pneumoniae 1). Median ICU length of stay was 14 (1;113) days.

**Table 2:** Main therapeutic interventions during ICU stay

**Therapeutic intervention**	
Noninvasive ventilation, n (%)	4 (13%)
Duration, days	4,5 (1–11)
Invasive ventilation, n (%)	25 (81%)
Duration, days	11 (1–109)
Vasopressive drugs, n (%)	14 (45%)
Duration, days	2,5 (2–27)
Inotropic drugs, n (%)	5 (16%)
Duration, days	5 (2–20)
RRT, n (%)	18 (58%)
Duration, days	3,5 (1–66)

Data are presented as median (range), otherwise specified*.*

*RRT* renal replacement therapy*.*

**Figure 1:**
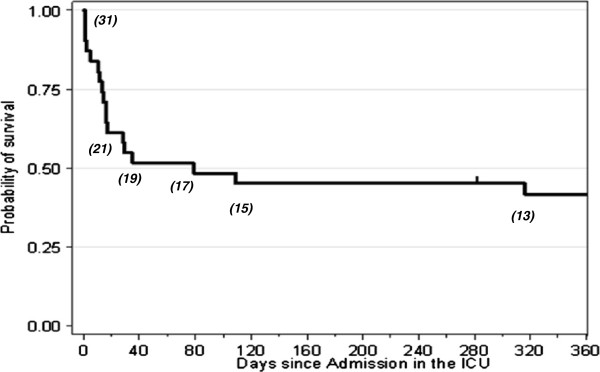
**Kaplan-Meier curve for one year survival after ICU admission of patients with vasculitis diseases.** The number of patients at different time points is displayed under the curve.

Sixteen patients (52%) died in ICU. This ICU mortality was significantly higher than that of the control group of ICU patients which was at 20%; differences between these two groups are displayed in Table 3. Reasons of death were septic shock (n = 7), refractory hypoxemia during ARDS (n = 7: infectious 3 and intraalveolar haemorrhage 4), ventricular fibrillation during myocardial infarction (n = 1) and fatal subarachnoidal haemorrhage (n = 1). Causes of death according to the vasculitis diagnosis are displayed in Table 4. Two patients died during the one-year follow-up which brings mortality to 58%. Figure 1 represents the Kaplan-Meier curve for one year-survival after admission to the ICU.

**Table 3:** Comparison between ICU control and ICU vasculitis patients

	***ICU control patients (31)***	***ICU vasculitis patients (31)***	***p***
*Age, yo*	*58 ± 12.8*	*61 ± 13.5*	*0.78*
*SAPS II*	*39.4 ± 16.6*	*42 ± 21*	*0.21*
*SOFA*	*5.6 ± 3.5*	*6.2 ± 4.1*	*0.9*
*Deceased, n (%)*	*6 (19.4)*	*16 (51.6)*	*0.001*

Data are presented as mean ± standard deviation.

**Table 4:** Causes of death according to vasculitis diagnosis

	**Deceased**	**Causes of death**	**n**
**Granulomatosis with polyangiitis (15)**	**9**	Septic shock	3
		Alveolar haemorrhage	3
		Respiratory infection	3
**Other vasculitis (16)**	**7**	Septic shock	4
		Alveolar haemorrhage	1
		Subarachnoidal haemorrhage	1
		Ventricular fibrillation	1

### Prognostic analysis of the 31 vasculitis patients admitted to the ICU

By univariate analysis, the comparison between survivors and non-survivors showed that ICU mortality was statistically associated with higher age (p = 0.04), SOFA (p = 0.006) and SAPS II (p = 0.004) scores (Table 5). There was a tendency that deceased patients had higher BVAS scores compared to survivors (p = 0.06). No differences in comorbities and prior health status were observed between surviving and non-surviving patients. During ICU course, the need for catecholamine or/and renal replacement therapy was also associated with ICU mortality. The occurrence of an ARDS worsened significantly the prognosis. ICU length of stay was similar between the two groups.

**Table 5:** Univariate analysis comparing ICU survivors and non-survivors

**n**	**Deceased (16)**	**Survived (15)**	**p**
Age, years	69,5 [29–86]	57 [32–78]	0,04
Sex ratio, m/f	12/4	9/6	0,45
BVAS	24.5 [12.0-52.0]	21.0 [2.0-28.0 ]	0,06
SAPS II	46.5 [21.0-109.0]	35.0 [13.0- 51.0]	0,004
Mechanical Ventilation, n (%)	15 (94%)	10 (67%)	0.08
SOFA	7.0 [2.0-15.0]	3.5 [1.0-10.0]	0.006
VAP, n (%)	10 (62.5%)	10 (66.7%)	0.80
Vasopressive agents, n (%)	13 (81,3%)	5 (33,3%)	0.006
ARDS, n (%)	13 (81,3%)	4 (26,7%)	0.002
RRT, n (%)	13 (81,3%)	5 (33,3%)	0.007
Creatininemia, microm/l	297 [56–1067.0]	76 [32–449]	0.062

Data are presented as median (range) or No/total patients.

*BVAS* Birmingham Vasculitis Activity Score*, SAPSII* simplified acute physiology score II*, SOFA* sequential organ failure assessment*, VAP* ventilator associated pneumonia*, ARDS* acute respiratory distress syndrome, *RRT* renal replacement therapy*.*

Among the variables from the univariate analysis included in the multivariate model, SAPS II and BVAS scores (at any given number) were independently related to the mortality. The adjusted odds ratio for the ICU mortality associated with BVAS and SAPS II were 1.16 (95% CI = [1.01- 1.34]) and 1.16 (95% CI = [1.01-1.33]) respectively. No other variable associated with mortality in the univariate analysis remained significant in the multivariate analysis.

## Discussion

The aim of the current study was to describe the epidemiology, the clinical features, and the outcome of vasculitis patients who were admitted to an ICU. The mortality rate for this population was 52% in the ICU and 58% one year later which was significantly higher than the mortality rate for a general ICU population (less than 20% in our ICU). Our results demonstrate also that SAPS II and BVAS scores were the main independent indicators of prognosis.

Systemic necrotizing vasculitis is a group of diseases defined by vessel wall inflammation that, although heterogeneous, shares some common clinical characteristics and therapeutic management. ICU admissions of such patients are mainly justified by the occurrence of severe active disease related-organ dysfunction or severe complication of immunosuppressive therapy, mostly infection. In this study, severe active manifestation of the disease was the reason of ICU admission in 61% of our patients. A pneumo-renal syndrome with both acute pulmonary and renal failure occurred in 9 patients and, as a manifestation of Granulomatosis with polyangiitis in 8 of them. Cruz and colleagues [6], including 26 patients with systemic necrotizing vasculitis, found also an active manifestation (77%) as a major reason of ICU. It is noteworthy in their report that vasculitis exacerbation was often the first manifestation of the disease leading to its diagnosis which was the case in 7 (23%) of our patients. In the study of Khan et al. [7], diffuse alveolar hemorrhage was the most frequent reason of ICU admission which is also an active manifestation of the disease. More recently, a severe alveolar haemorrhage was reported as the first disease manifestation in 46 (86.8%) of 53 patients with ANCA-associated vasculitis [23]. Infections represent the second cause of ICU admission [7] as in ours. Eight (26%) patients presented indeed a septic shock requiring critical care management. The well recognized risk of infection in these patients is increased [4]; and is determined primarily by the level of immunosuppression. Actually, all the 8 patients admitted for severe sepsis received more than 20 mg steroids daily at least in the last three months. Godeau et al. [8] showed, in a study including 181 patients with systemic rheumatic disease admitted to ICU that, in the sub-group of 39 vasculitis, the admission infection rate was at 49%. However, the immunosuppressive treatment was not reported precisely.

ICU severity scores and critical care management of our population underlined its high morbidity. More than ¾ of patients were on mechanical ventilation, more than half required vasopressive or inotropic drugs and renal replacement therapy. For comparison, Khan [7] found that 45% of patients were ventilated and 29% underwent hemodialysis. The ICU mortality in our cohort was 52% which is higher than that of a general ICU population [9]. This high ICU mortality rate underscores the pejorative role of the specific disease and/or of long term immunosuppressive treatment. Studies focusing on outcome and prognosis of patients with vasculitis admitted to ICU are scarce. They include mostly systemic rheumatic diseases like connectivitis, rheumatoïd polyarthritis, granulomatosis, primary vasculitis and systemic vasculitis [10-12]. To the best of our knowledge, only *3* studies investigated specifically vasculitis ICU outcome and reported lower mortality rates varying from 11 to 33,3% [6,7,13]. However, these results should be interpreted with caution, at least under the light of the severity of both the specific disease and the associated organ failure. As earlier notified, 80% of our patients were mechanically ventilated with severe respiratory disease and patients with pulmonary vasculitis were reported to have an ICU mortality of more than 50% depending on the disease severity [15,24,25]. Last, we observed that two patients among survivors deceased one year after they were discharged from hospital underlying that the occurrence of severe complications requiring an ICU admission may also affect long term survival [15]. Previous studies reported similarly a significant increase in mortality at a one year (from 11 to 29%) [7], and a 30 months (from 15 to 39%) [6] follow-up. In the recent report of Hruskova and colleagues [23], the long-term prognosis of patients with severe ANCA-associated vasculitis showed that the mortality increased from 17% at 3 months to 41.5% at 49 months median follow-up.

The identification of predictive factors for ICU mortality in such patients is obviously an important matter to study, since it may help in an optimal follow-up and management. Several factors have been previously reported to be predictive of ICU mortality [16,17]. We identified SOFA and SAPS II severity scores, the need for catecholamine or/and renal replacement therapy and the occurrence of an ARDS as prognosis factors by univariate analysis. However including these parameters in a multivariate analysis, only SAPS II and BVAS scores were associated with an increased risk of ICU mortality. ICU gravity scores especially SAPS II and SOFA scores were previously reported as predictive factors of mortality for vasculitis in ICU in several studies [6,7,13]. By contrast, BVAS score was not found to be correlated to ICU mortality [6,7,13]. Cruz and colleagues [6] observed similar BVAS scores in survivors and non survivors (16,4±10.3 vs 15.8±20.1; p = 0.75) in a 30- month follow-up. Khan et al. [7], using a modified BVAS/WG score adapted to Granulomatosis with polyangiitis, also failed to correlate it with a 28 day ICU mortality. Frausova and al. [13] reported even a higher, although not significant, BVAS in survivors (16.1 vs 11.5). Our observation need to be confirmed but highly suggests that vasculitis disease activity influence short-term prognosis. Cruz et al. [6] found however that BVAS calculated at ICU admission was significantly higher in non-survivors than survivors at the end of a long-term follow-up of patients with active systemic necrotising vasculitis.

We must acknowledge some limitations to our study. First, it was limited to 4 ICUs from 2 university hospitals and consequently its findings should not be generalized. The sample size is, in addition, relatively small and heterogeneous given the rarity of these diseases. Second, it is a retrospective study which may therefore be affected by various biases. Third, immunosuppressive treatments had changed between individuals which may represent additional bias in the interpretation of the results.

## Conclusion

In summary, severe vasculitis complications requiring admission to the ICU are mostly of active disease manifestation or infectious origin. ICU mortality is significantly higher underlying the role of intensive or long term immunosuppression on worsening the outcome. SAPS II and BVAS scores upon ICU admission are predictive factors of mortality and may be helpful to early identify high risk patients.

## Consent

Written informed consent was obtained from the patient or his closed relative for the publication of this report and any accompanying images.

## Competing interests

The authors declare that they have no competing interests.

## Authors’ contributions

KK, PB, PC and OJ conceived the study, created its design, collected the data and drafted the manuscript. TF performed the statistical analysis. BJ, CB, PB and KK participated in collecting the data. All authors read and approved the final manuscript.

## Pre-publication history

The pre-publication history for this paper can be accessed here:

http://www.biomedcentral.com/1471-2253/13/27/prepub
